# Evaluating the generalizability of graph neural networks for predicting collision cross section

**DOI:** 10.1186/s13321-024-00899-w

**Published:** 2024-08-29

**Authors:** Chloe Engler Hart, António José Preto, Shaurya Chanana, David Healey, Tobias Kind, Daniel Domingo-Fernández

**Affiliations:** Enveda Biosciences, Inc., 5700 Flatiron Pkwy, Boulder, CO 80301 USA

## Abstract

Ion Mobility coupled with Mass Spectrometry (IM-MS) is a promising analytical technique that enhances molecular characterization by measuring collision cross-section (CCS) values, which are indicative of the molecular size and shape. However, the effective application of CCS values in structural analysis is still constrained by the limited availability of experimental data, necessitating the development of accurate machine learning (ML) models for in silico predictions. In this study, we evaluated state-of-the-art Graph Neural Networks (GNNs), trained to predict CCS values using the largest publicly available dataset to date. Although our results confirm the high accuracy of these models within chemical spaces similar to their training environments, their performance significantly declines when applied to structurally novel regions. This discrepancy raises concerns about the reliability of in silico CCS predictions and underscores the need for releasing further publicly available CCS datasets. To mitigate this, we introduce Mol2CCS which demonstrates how generalization can be partially improved by extending models to account for additional features such as molecular fingerprints, descriptors, and the molecule types. Lastly, we also show how confidence models can support by enhancing the reliability of the CCS estimates.

**Scientific contribution**

We have benchmarked state-of-the-art graph neural networks for predicting collision cross section. Our work highlights the accuracy of these models when trained and predicted in similar chemical spaces, but also how their accuracy drops when evaluated in structurally novel regions. Lastly, we conclude by presenting potential approaches to mitigate this issue.

**Supplementary Information:**

The online version contains supplementary material available at 10.1186/s13321-024-00899-w.

## Introduction

Ion Mobility coupled to Mass Spectrometry (IM-MS) has emerged as a powerful analytical technique that complements traditional mass spectrometry by providing additional insight into the structural properties of ions [7]. IM-MS measures the time ions take to traverse a gas-filled chamber under an electric field. The drift time is then used to calculate the collision cross section (CCS) of the ions. Since CCS is a reproducible and structure-reflective parameter that characterizes the overall shape and size of ionized molecules, it can be used as an orthogonal feature to identify the compounds in a sample. Therefore, leveraging CCS data can be viewed as a complementary approach to the traditional liquid chromatography mass spectrometry (LC–MS) based approaches, enhancing the accuracy and depth of structural prediction approaches [4].

The benefits of using CCS values for structure prediction are evident by the growing number of related publications, but various challenges hinder their wider adoption. A major challenge is the limited availability of comprehensive CCS databases, which often lack extensive coverage of CCS values for a wide array of reference compounds [13, 17, 23]. While many of the databases cover a wide range of predicted values, the number of high quality experimental references is only in the low thousands.

This deficiency significantly restricts the effective use of CCS as a predictive tool in structural analysis. To account for the lack of experimental CCS values, in silico prediction models can be used. The efficacy of these predictions, however, is contingent upon the precision of these models and the used structural scaffolds during model training. In other words, only models that can reliably predict CCS values with high accuracy are suitable for generating synthetic CCS values that can fill the gaps in experimental values existing in current databases. The concept of the applicability domain is well known in the field of QSAR/QSPR and property predictions [5, 18]. It refers to the concept that both training and validation compounds and their estimated parameters need to be in a similar structural space (scaffold space) and that predicted properties should be similar in the training and prediction set (response space). This ensures that the models perform reliable, robust and that confident predictions can be made and outliers could be marked as potentially unreliable predictions [10, 11]. Without such advancements, the integration of CCS as a routine parameter in molecular characterization remains underutilized, limiting our capacity to fully exploit its insights into molecular geometry and interactions.

Over the past few years, several machine learning (ML) models for predicting CCS values have been developed [9]. These models require a molecular representation, such as fingerprints or a graph representation of the molecule. The first ML model, DeepCCS [14], encodes SMILES representations and utilizes a convolutional neural network to predict CCS values. It was trained and evaluated on a set of heterogeneous datasets containing over 2,400 molecules combined. Similarly, a Support Vector Regression (SVR) model named CCS Predictor 2.0 [16] leverages molecular fingerprints to predict CCS values and has demonstrated better accuracy than DeepCCS.

More recently, two models based on Graph Neural Networks (GNNs) have surpassed previous state-of-the-art (SOTA) models in predicting CCS values using a graph representation of molecular structures. The first model, SigmaCCS [6], employs Edge-Conditioned Convolutions (ECCs) [19] to embed the original molecular structure along with the adduct type. The authors evaluated the model on CCSBase (v1.2) [17], focusing on over 5,000 high-quality experimental CCS values and three adducts ([M + H] + , [M + Na] + , and [M-H] − . They reported a coefficient of determination (R^2^) of 0.9945 and a Median Relative Error (MRE) of 1.1751% on a test set. The second GNN, GraphCCS [22], simulates the structure of the resulting adduct and uses it as input for a Graph Convolutional Network (GCN). Although these models have not been directly compared, Xie and colleagues also reported an R^2^ of 0.994 and an MRE of 1.29% on a different test set from CCSBase (v1.2). Lastly, both studies have used their models to generate an in silico database of CCS values.

Until recently, CCSBase [17], and its underlying datasets, was the main publicly available source where researchers could access several thousand CCS data points. Consequently, any prior modeling approach has been constrained by the limited amount of training data available. Notably, although previous evaluations have demonstrated that models can accurately predict CCS values from molecular structures, the chemical space represented in this database is relatively small (approximately 6,075 unique structures, including lipids, peptides, carbohydrates, and small molecules) and highly homogenous (see Supplementary Fig. 1A). The recent release of METLIN-CCS [1, 2], which focuses on synthetic small molecules, significantly expands the availability of experimental CCS data with over 27,000 unique structures. This expansion allows for the benchmarking of previously published models in a broader context. Additionally, the combination of fingerprints and GNNs has recently been applied to similar prediction tasks, such as retention time prediction [22]. Furthermore, leveraging additional metadata, such as the instruments used to generate the CCS values or the types of molecules analyzed, could potentially enhance the accuracy and generalizability of these models.

In this work we benchmark the state-of-the-art models on the new METLIN-CCS database and assess their generalizability. We also demonstrate increased generalizability using an extension of SigmaCCS, which we call Mol2CCS, that incorporates additional information such as instrument type. Finally, we show that using confidence models to filter predictions can result in increased performance of the models.

## Methods

### Data

For this work, we leveraged two of the largest public resources for CCS values: CCSBase [17] and METLIN-CCS [1]. CCSBase (v1.3) combines 22 different datasets, providing a total of 16,989 CCS values measured on three different instruments from 6,744 distinct molecules (e.g., small molecules, lipids, peptides, and carbohydrates). Recently published, METLIN-CCS (downloaded on 14/04/2024) is currently the largest CCS database with over 65,000 CCS values from 27,633 distinct small synthetic molecules. METLIN-CCS contains CCS values exclusively measured in timsTOF Pro for trapped ion mobility spectrometry (TIMS) and CCS values for individual adduct forms were experimentally acquired in triplicates.

### Pre-processing

#### SMILES

There are multiple ways of representing small molecules, one of the most common ones being SMILES codes. This format allows encoding the molecular data into string format, without loss of information. In this analysis, the SMILES strings available in both datasets are the base representation leveraged to generate molecular fingerprints and graph representations.

#### Standardization across datasets

Handling adduct ions is a common hurdle when dealing with mass spectrometry (MS) data [20]. As a byproduct of the measurement, adducts become a source of variation that needs to be considered in order to account for the same molecule displaying different CCS values. Both GraphCCS and SigmaCCS take this into account by passing adducts as features, in our work, we decided to also include it in our data splitting schema in order to make sure that the same molecule with different adducts does not get separated across the prediction model training and evaluation stages. Similarly to previous work, we considered only the most common adducts. Since this work is the first to leverage the recently released METLIN-CCS for CCS prediction, we are able to train models on one order of magnitude more than previous work, we considered more adduct forms (9) than previous work (3): [M + H] + , [2 M + H] + , [M + Na] + , [2 M + Na] + , [M-H]-, [2 M-H]-, [M + K] + , [M + H-H2O] + , [M + NH4] + .

Due to the diversity of CCSBase and METLIN-CCS, we followed similar processing steps as GraphCCS and SigmaCCS. We first dropped rows with missing SMILES and SMILES with a “.”, implying multiple disconnected parts. In CCSBase multiple instances of duplicate SMILES-adduct pairs were present. To address this issue, we calculated the standard deviation of the CCS values associated with each SMILES-adduct pair. We removed any pairs with a mean absolute deviation greater than five, determined through analysis of a histogram showcasing all mean absolute deviations for duplicates (Supplementary Fig. 2). We then assigned the mean CCS value for each pair to the remaining duplicates. Ultimately, these processing steps reduced the number of data points from 16,989 to 13,617 for CCSBase. All points within the METLIN-CCS database had a mean absolute deviation less than five.

#### Feature extraction for Mol2CCS

Similarly to related work [6, 22], the Mol2CCS architecture represents each molecule as a graph, wherein every node and edge denoted an atom and bond, respectively. The entire molecular graph is represented by three different matrices: i) node attribute matrix, ii) edge attribute matrix, and iii) adjacency matrix. These three matrices respectively store characteristic attributes of the atoms, bonds, and the connections of the molecular graph. To construct them, we first read the SMILES representation of each molecule using RDKit (v2023.09.5) [8] and subsequently used the ETKDG and MMFF94 conformer generators to obtain the 3D conformers for each molecule. Lastly, from these conformers, we obtained the atoms, bonds and their attributes. As features for Mol2CCS, we expand upon the node and bond attributes used by Guo et al. [6] (Supplementary Table 1).

In addition to the molecular graph and the adduct, Mol2CCS utilizes seven additional features encoded as one-dimensional vectors. Firstly, similar to previous models, we applied one-hot encoding to represent specific adduct forms (i). Additionally, we employed one-hot encoding to differentiate between monomeric and dimeric adducts (ii). Secondly, we integrated molecular information by incorporating a 256-dimensional vector representing Morgan fingerprints (iii), generated using RDKit with 256 bits and radius of 2, and a 2-dimensional vector containing the molecular weight of the original molecule as well as the molecular weight of the original molecular plus or minus the adduct (iv). Thirdly, to specify the type of molecule, we included a 35-dimensional vector indicating the presence or absence of several structural classes (v) (e.g., allene, carboxyl, and organic acid) using Drug Tax [15]. Additionally, we applied one-hot encoding to categorize the four molecule types (vi) (i.e., small molecule, lipid, peptide, carbohydrate). Lastly, we incorporated a final one-hot encoded vector indicating the instrument type (vii) (i.e., TIMS, DT, TW).

### Data splitting

**Fig. 1:**
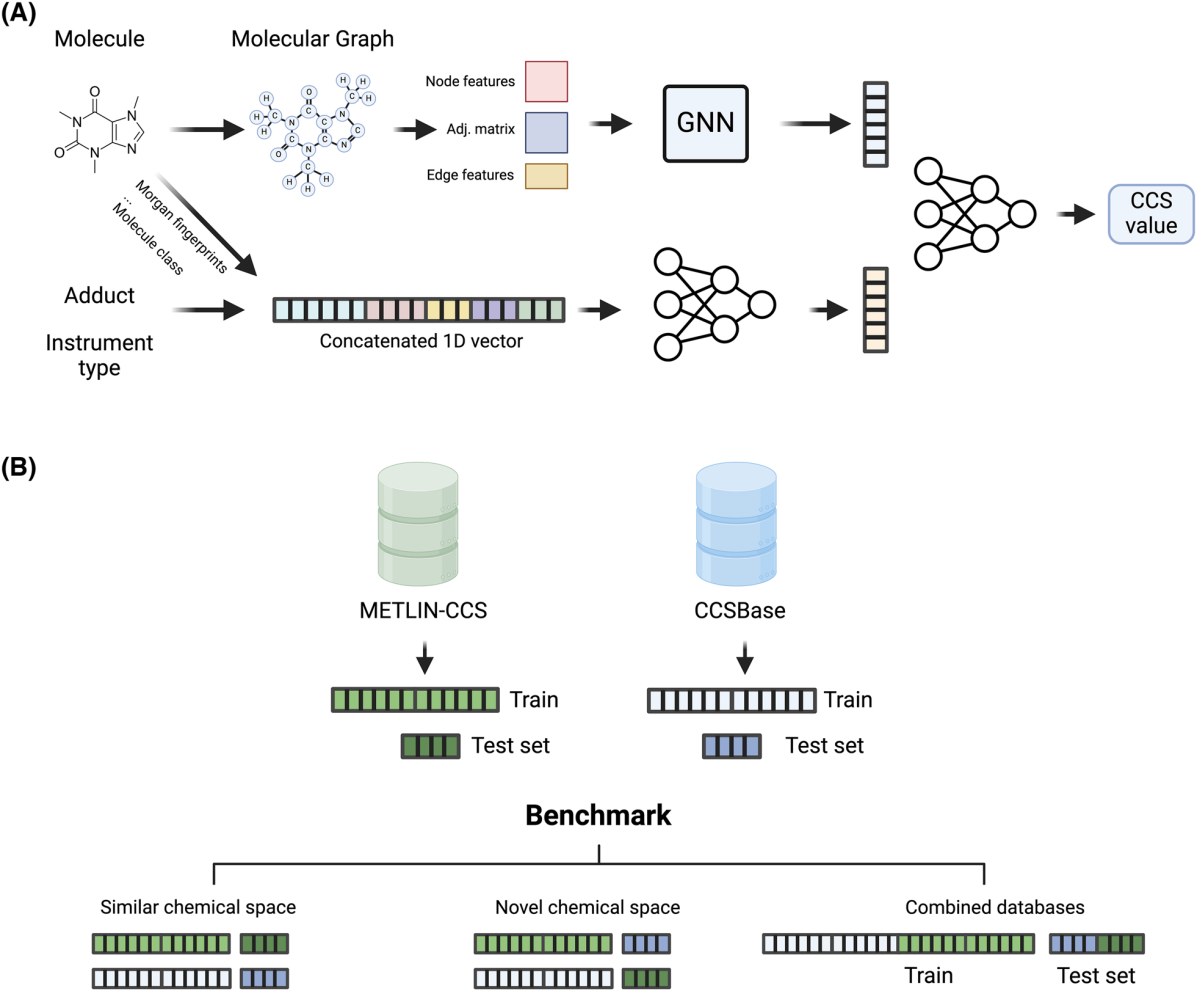
**A** Model architecture. The upper section of this figure illustrates the conversion of the SMILES representation of the molecule into a molecular graph, which is then represented as three matrices (an adjacency matrix, an edge attributes matrix, and a node attributes matrix). These matrices are fed into a GNN. The GNN’s output is concatenated with the output from a linear model which accepts additional features (such as adduct, instrument type, etc.) as input. This concatenated vector is then fed into another set of fully connected layers which outputs a CCS value. **B** Evaluation schema. Each database is split in train (80%) and test (20%) based on molecule type (e.g., lipid, small molecule, etc.) and Murcko scaffolds. Next, each model is trained on the training set of each database (either CCSBase train or METLIN-CCS train) and evaluated on the two test sets of both databases (CCSBase test and METLIN-CCS test). When the model is evaluated on the same database that has been trained on, the model has already seen similar molecules, and thus, the evaluation is on similar chemical space (left). When the model is evaluated on a test set containing dissimilar molecules, the evaluation is a novel chemical space (middle). Lastly, both databases are also combined for training and testing (right)

We trained and tested each model on train and test splits using the following strategy. First, we divided the entire dataset (CCSBase and METLIN-CCS data combined) into five distinct groups: lipids, dimers carbohydrates, peptides, and everything else. This categorization ensured that each of these categories was represented in the train and test sets. We performed an 80/20 train test split on each of these groups stratified on the Murcko scaffold [3]. It is important to note that several molecules did not have Murcko scaffolds (mostly lipids) and some of them only a simple benzene Murcko scaffold. Given the substantial number of molecules in these two categories, we chose to replace the Murcko scaffold with the SMILES string during the stratified split for such molecules. After performing these splits, we combined all of the train splits and all of the test splits to create our training and test sets. There were several Murcko scaffolds that appeared in more than one of our initial five groups, resulting in 745 scaffolds that were present in both the training and test sets. To ensure disjoint testing and training sets, we performed an additional 80/20 train/test split on these 745 scaffolds to divide them into the train and test sets (see Supplementary Fig. 3 for details). For models that were trained solely on a single database (i.e., METLIN-CCS or CCSBase), we used these same data splits confined to that particular database **(**Fig. 1B**)**. This method ensured that the test data for the combined dataset didn’t contain any molecules used for training models on the METLIN-CCS or CCSBase data alone.

### State-of-the-art machine learning models for CCS prediction

We evaluated the performance of the two most accurate ML models trained to predict CCS values: GraphCCS [22], and SigmaCCS [6]. We retrained these models using the reported hyperparameters on the datasets used in this work.

### Mol2CCS’s architecture and hyper-parameters

Since Mol2CCS is an extension of SigmaCCS [6], we expanded upon its codebase for its implementation. Our goal, rather than developing a new model, was to demonstrate how current SOTA models can be improved by leveraging additional features, similar to [22] for retention prediction.

The SigmaCCS architecture consists of a GNN with three ECC layers [19] trained on three matrices representing the molecular graph described in Section"Feature extraction for Mol2CCS". The output of these layers is concatenated with a one-hot encoded vector representing the adduct and fed into several fully connected layers to produce a predicted ccs value. In our implementation of Mol2CCS, the GNN module is identical to SigmaCCS’s implementation, but we extend the model with a parallel module consisting of four fully connected layers (# neurons: 256, 512, 512, 256) applying dropout to prevent overfitting. This module learns a representation for the additional seven features (including adduct) (see subsection "Feature extraction for Mol2CCS"). Lastly, the model aggregates the output of both modules into eight fully connected layers with 384 neurons each, as the original SigmaCCS model (Fig. 1A). For both the ECC layers and the fully connected layers, we used ReLU as activation functions and the L2 regularization. The final layer that outputs the predicted CCS value is also a fully connected layer with ReLU but without regularization applied.

We trained the model up to 400 epochs applying early stopping using a patience of 10 epochs. Furthermore, we used a dropout of 0.1 for the novel module, a batch size of 32, an Adam optimizer with a learning rate of 0.0001, and 16, 16 and 128, respectively, as the outputs of the three ECC layers. We chose these parameters based on a grid search experiment conducted on a subset of the dataset (Supplementary Table 2). Details about the hardware used can be found in Supplementary Text 1.

### Evaluation

We benchmarked all the models using three different settings. In the first setting, we trained each model on 80% of one database (METLIN-CCS or CCSBase) and tested it on the remaining 20% of the same database. This method evaluated the model’s ability to predict CCS values for chemicals within a similar chemical space. For the second method we trained the model on the training set for one database and used the other database as a test set. The rationale behind this setting was to evaluate the generalizability of both datasets and models. Finally, we trained both models on a combined dataset using both METLIN-CCS and CCSBase (Fig. 1B). Supplementary Table 3 and 4 report the number of adducts and molecule types in each database.

We evaluated the performance of the models on a test set using several metrics. We employed correlation metrics such as coefficient of determination (R^2^), Pearson and Spearman correlation. Additionally, we used mean absolute error (MAE), mean squared error (MSE), root mean squared error (RMSE), relative standard deviation (RSD), and the relative error in percentage between the predicted CCS values and the experimental ones (% CCS error).

### Confidence model

For our confidence model, we used a random forest model implemented using scikit-learn [12] (Supplementary Text 2). The model inputs for each molecule consisted of the seven features described above used for the novel module of Mol2CCS (e.g., SMILES, adduct, molecule type, CCS instrument type, etc.) as well as the experimental and predicted CCS values. To feed the features into the model, we calculated the Morgan fingerprints using radius = 2 and default values in RDKit from the SMILES string and one-hot encoded the remaining features. To create the labels for this model, we calculated the difference between the true CCS value and the predicted CCS value. If this difference was less than 5% of the true CCS value, we gave the molecule a label of one (i.e., a proxy for an accurate prediction), otherwise a label zero (i.e., a proxy for an inaccurate prediction). We then used the model’s predicted probabilities for our confidence scores. These confidence models were used for the models trained on one database (database A) and tested on another (database B) as an attempt to improve generalizability. To train the confidence models, we experimented with several training methods. For each of these methods, we used the original test set for database B to test the confidence models. Additionally, we sampled 10% of the original training set for database B to use as a validation set for determining confidence thresholds. We used the following training sets for the confidence model:

- A training set in the same domain as the training set for the CCS prediction model (the original test set for database A). This is equivalent to evaluating the confidence model in a different chemical space.
- The test set for database A with small amounts of data from the domain of database B (sampled from the original training set for database B excluding the validation set). This is equivalent to evaluating the confidence model in a different chemical space while exposing the model to a few in-domain chemical structures.
- A training set in the same domain as the test set (the original training set for database B excluding the validation set). This is equivalent to evaluating the confidence model in a similar chemical space.

To select the confidence thresholds (to filter out points with low confidence), we calculated the precision and recall for each threshold from 0 to 1 (with step size 0.1) and selected the highest threshold where the recall remained higher than the precision to balance recall and precision performance. This threshold varied for the different training sets.

## Results

### Benchmarking models on a similar chemical space

**Fig. 2:**
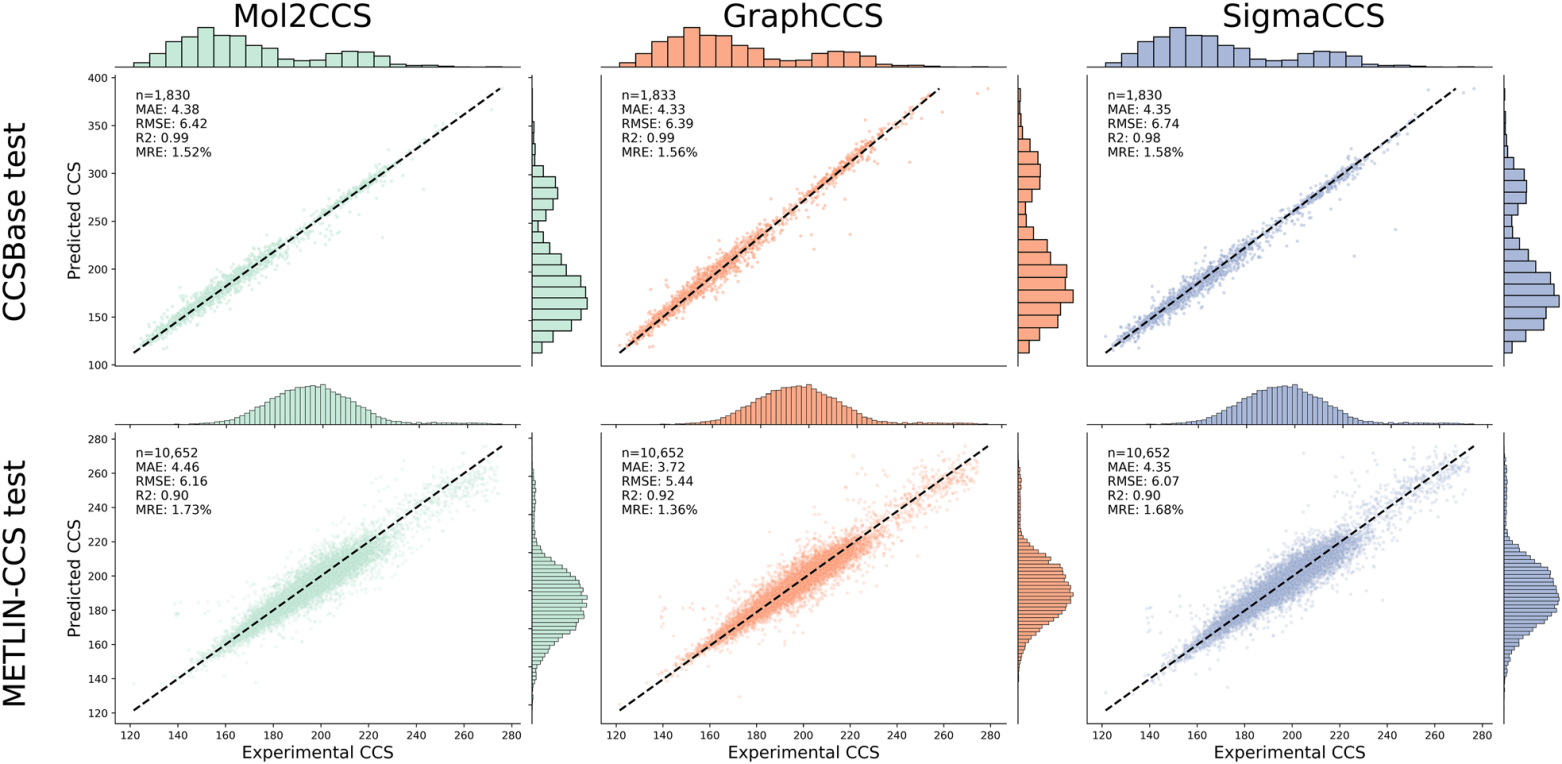
Scatterplots of the predictions for each model when training and evaluating on the same database. On the CCSBase dataset (upper row) all models perform equally with a very high correlation coefficient and RMSE of approximately 6 square angstrom [Å^2^]. On the METLIN-CCS, R^2^ drops from 0.99 in CCSBase to 0.9. However, the other metrics are comparable for all three models

We began by assessing the performance of the models in a similar chemical space by training them and evaluating them in the same database, either METLIN-CCS or CCSBase, independently. We would like to note that although we applied scaffold splitting rather than a simple train-test split to avoid data leakage, both databases comprise highly similar molecules (Supplementary Fig. 1C-D). Figure 2 shows the predicted CCS and the actual experimental values as well as the corresponding metrics for the three benchmarked models, when they are evaluated on a test set of the same database where they were trained.

Overall, the performance in both databases across all three models was very high. Although the performance on the CCSBase dataset is slightly worse than reported by either GraphCCS or SigmaCCS, presumably due to the scaffold splitting conducted, both models achieve a R^2^ slightly below 0.986, and a Median Relative Error (MRE) of 1.55% and 1.57%, respectively **(**Fig. 2**)**. Mol2CCS exhibits high accuracy across all metrics (e.g., R^2^ = 0.985, MRE = 1.51%, and MAE = 4.37). Similarly, the performance is also high for METLIN-CCS, with similar values for RMSE, MAE, and MRE, although R^2^ drops to 0.9 **(**Fig. 2**)**. Since this resource has a broader chemical space and molecules it contains are less similar between them, compared to CCSBase (where the models suffer from data leakage) (Supplementary Fig. 1), we believe the metrics for METLIN-CCS can accurately represent the performance of a model when it is evaluated on a similar chemical space. Finally, we investigated the molecules with the largest deviations between the predicted CCS value and the experimental one for each model and observed a high overlap, suggesting the outliers across all three models typically correspond to the same molecules (Supplementary Fig. 4).

### Benchmarking models on novel chemical space

Here, we explore the generalizability of the models when training them on one database (i.e., CCSBase and METLIN-CCS) and being evaluated on another one. Given that both databases cover different regions of the chemical space (Supplementary Fig. 5 and 6), these results can be used as a proxy to assess the performance of a model in a more realistic application, when the model has not seen closely similar molecules to the ones it is to predict. In this setting, the performance significantly drops across all models; thus, indicating the lack of generality of the models when predicting within an unseen chemical space.

**Fig. 3:**
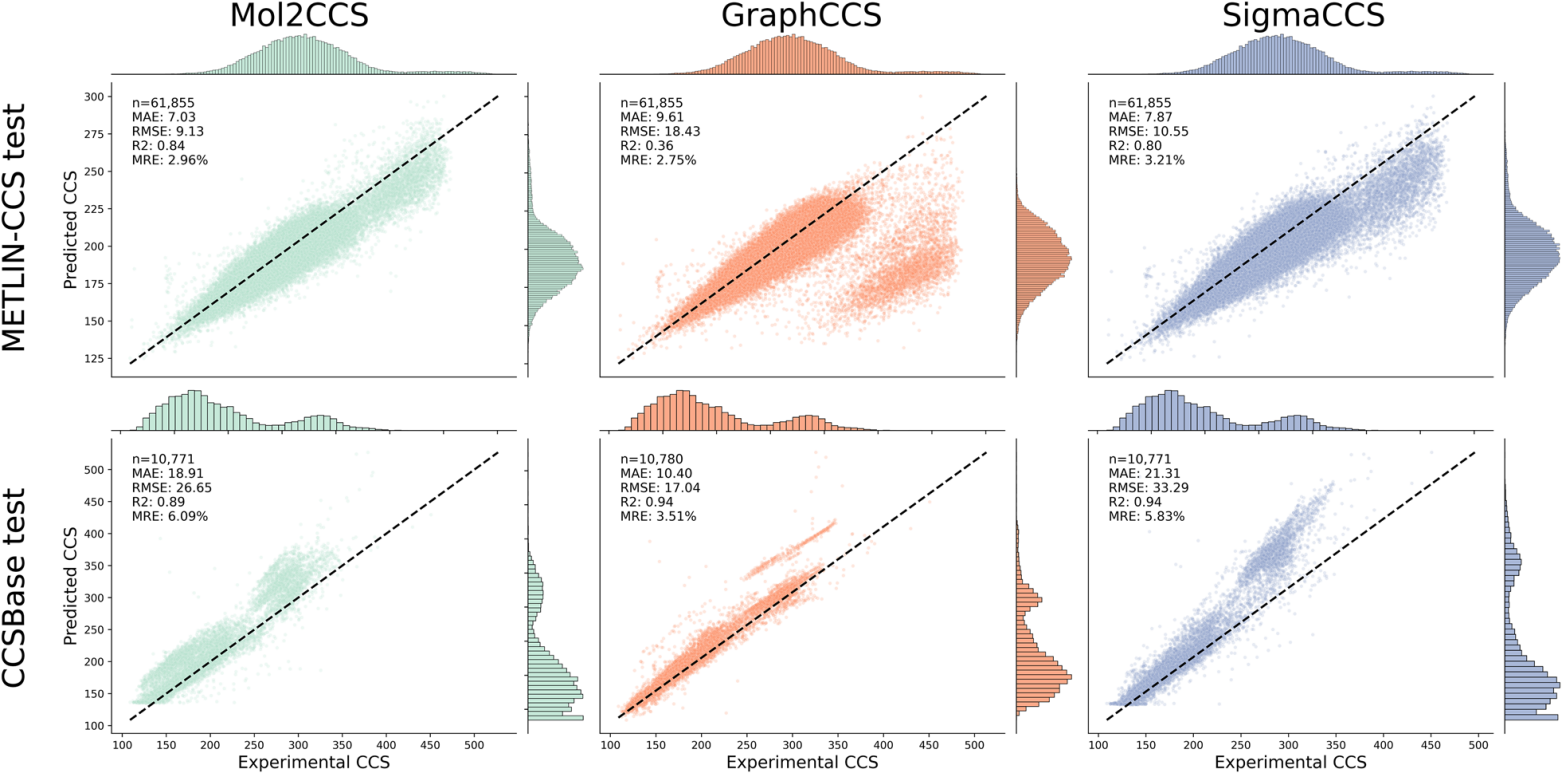
Scatterplots of the predictions for each model when training on one database and evaluating on another one. The bottom plots show the evaluation on CCSBase when training on METLIN-CCS. When training on CCSBase and evaluating on METLIN-CCS (upper row) the performance of all models significantly drops. For instance, the R^2^ goes down to 0.36, 0.8, and 0.84 for GraphCCS, SigmaCCS, and Mol2CCS, respectively. However, performance drops less dramatically when training on METLIN-CCS and evaluating on CCSBase, since the models have been trained on several times more data points. Despite the larger training data, the differences in their chemical space can explain why all models exhibit RMSEs three times larger than when they are trained and evaluated on the same database

When training on the CCSBase train set and evaluating on the entire METLIN-CCS **(**Fig. 3—top row), SigmaCCS and Mol2CCS drop to R^2^ lower than 0.8 and their RMSE, MAE and MSE are several times larger in comparison to the CCSBase test set. GraphCCS also performs poorly (R^2^ = 0.36, RMSE = 18.43, MAE = 9.61), due to larger errors predicting dimers (large cloud of orange points deviated from the diagonal). We would like to note that these inaccuracies for dimers are to be expected due to the limited number of dimer examples in the training set (CCSBase) (Supplementary Table 4). When we subset the test set to only monomers the models tend to perform better (i.e., GraphCCS shows better performance when evaluating solely based on the [M + H] + and [M-H]- adducts) (Supplementary Fig. 7). Likewise, when we trained on METLIN-CCS, which has more dimers, and evaluated on CCSBase, GraphCCS significantly improved its performance on these less common adducts. Overall, Mol2CCS achieves the best performance, as the additional features (e.g., molecular fingerprints, dimer type, molecule type, etc.) added to the model on top of SigmaCCS, lead to a better generalization of the model.

We observed a similar trend when training on the METLIN-CCS train set and evaluating on the entire CCSBase **(**Fig. 3—bottom row). While they all achieve a better performance in this setting (R^2^ above 0.89, RMSE between 17.04 and 33.29, MREs between 3.44 and 5.80%, and MAEs between 10 and 21), boosted by being trained on a larger and broader dataset, the performance is still worse than training and evaluating on a single database. When looking at GraphCCS, we observed again that there is a straight cloud of points deviated from the diagonal, which corresponds to the lipids in CCSBase. The same lipids, however, are more dispersed in both Mol2CCS and SigmaCCS.

### Evaluating the performance on both databases

**Fig. 4:**
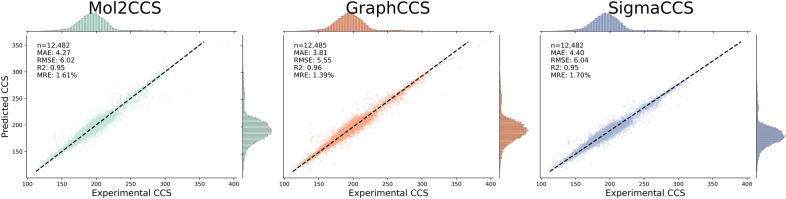
Scatterplots of the predictions for each model when training and evaluating on the combined dataset

As a final experiment, we trained all three models on the combined dataset comprising both databases. As expected for a model evaluated on a similar chemical space than the one it was trained on, the performance of the three models is high, lying between the metrics observed for in subSect. "Benchmarking models on a similar chemical space" for each database (Fig. 4**)**. All three models have a R^2^ close to 0.95, RMSEs close to 6, and MREs between 1.39% and 1.71%. Mol2CCS minimally improves the performance of SigmaCCS, suggesting that expanding the GNN architecture can also help when models are trained on large datasets and are evaluated on structurally similar compounds to the training data. The best model among the three is GraphCCS, which now achieves a good performance across all nine adducts, closely followed by the other two (Supplementary Fig. 8).

### Confidence models can assist in identifying high confidence predictions

In this subsection, we explore the use of confidence models to enhance CCS prediction models by flagging predictions likely to deviate beyond a predefined threshold. Specifically, we focused on predictions for novel chemical spaces using Mol2CCS (see SubSect. "Benchmarking models on novel chemical space"), where we observed high variability and numerous outliers in the predictions. Consequently, we set a 5% threshold for training the confidence model. It is important to note that other models or datasets could potentially be used for this task.

**Fig. 5:**
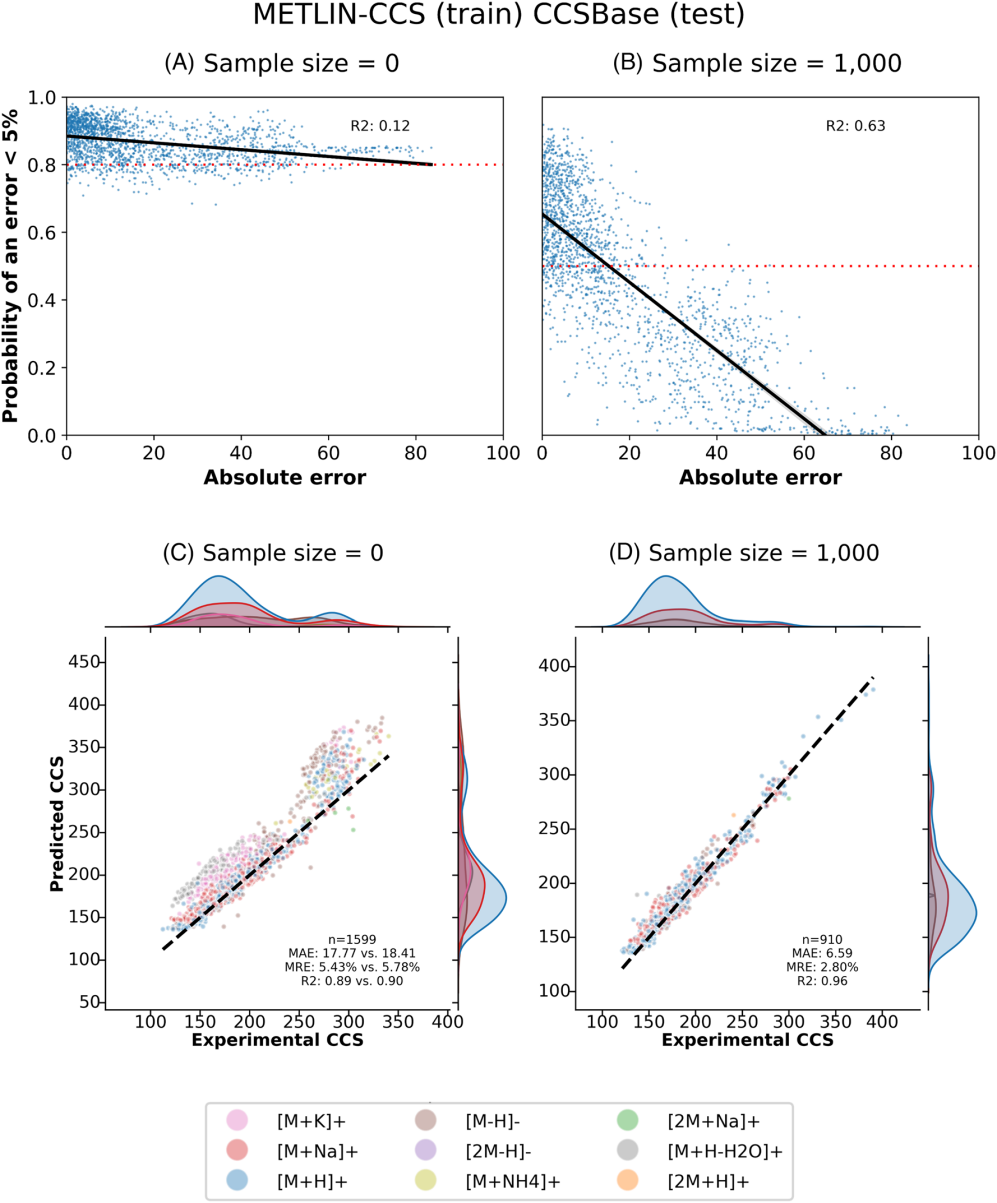
Confidence model for CCS prediction model trained on METLIN-CCS and tested on CCSBase. **A**-**B** Predicted confidences by the confidence model in the test set vs. absolute error of the Mol2CCS prediction. **C**-**D** Predictions on the high confidence subset generated from the two experiments where the model is trained on one database and evaluated on the other. **A** and **C** are for the confidence model trained only on data from METLIN-CCS. **B** and **D** show results for the confidence model trained on METLIN-CCS data with an additional 1,000 data points from METLIN-CCS (that are structure disjoint from the METLIN-CCS test dataset). **C** displays the metrics of the data after confidence thresholding compared to the metrics without filtering. Comparing **A** and **B** as well as **C** and **D** demonstrates that the confidence model improves when it is trained with some in domain data. However, as shown in **C**, even without in domain data, the MAE and MRE improve slightly when confidence thresholding is used

Initially, we examined the performance of the confidence model trained using data from the same domain as the training dataset used for the CCS prediction model. With this training set, the confidence model was very confident since all of the predictions it was trained on were relatively accurate (Fig. 2). When we filtered the compounds based on their confidence score, applying a threshold (0.8) determined by the methods described in Sect. "Confidence model", the metrics remained almost unchanged. Nevertheless, MAE and MRE did decrease slightly (Fig. 5C and Supplementary Fig. 9C).

Next, we tried enhancing the confidence model train set with small amounts of scaffold disjoint data from the test set domain. We examined the MAE, MRE, and R^2^ metrics for sample sizes of 5, 10, 25, 50, 100, and 1,000 (Supplementary Fig. 10). As the sample sizes increased, the MAE and MRE decreased for both the models illustrating that the confidence model was able to remove outliers (Supplementary Fig. 11). Additionally, the correlation between the probabilities of an accurate prediction and the actual performance of the confidence model continued to increase, indicating the confidence model is able to accurately predict the CCS values that are off by over 5% (Fig. 5A and B). Interestingly, the model trained on CCSBase exhibited less overall improvement. This is likely because the CCSBase dataset contains several molecule types (such as lipids) that the METLIN-CCS database does not. So when the confidence model is trained exclusively on METLIN-CCS data and tested on CCSBase data, it has no reference point for these other molecule types. However, when we add a small amount of CCSBase data to the confidence model training set, the confidence model can better handle these out of domain molecule types on the CCSBase test set causing a significant improvement in the metrics. To summarize, these results demonstrate that even if there is only a small amount of in-domain training data available, this data can be used to train confidence models with better performance.

Finally, we examined the performance of the confidence model trained on only data from the same domain as the test set (Supplementary Fig. 12). Using this training set, the MRE and MAE metrics were consistent with those for the confidence model trained with 1,000 in domain data points described above. Still, the correlation between the probabilities of an accurate prediction and the actual performance of the confidence model increased particularly for the model trained on CCSBase. This increased correlation may have had a larger impact on the MRE and MAE a different confidence threshold (Supplementary Fig. 13). Nevertheless, this result further demonstrates that more in-domain data enables confidence models to more accurately identify outliers.

In conclusion, each of these confidence models demonstrated to varying degrees that focusing on the set of high confidence predictions can increase model performance. This is particularly true when even a small amount of in-domain data is included in the training set for the confidence model.

## Discussion

In this work, we evaluated the performance of state-of-the-art deep learning models for predicting CCS values from molecular structures and proposed two novel modeling approaches that could be implemented to improve accuracy and confidence. First, we benchmarked two GNNs (i.e., SigmaCCS and GraphCCS) and Mol2CCS, an adaptation of SigmaCCS, on METLIN-CCS and CCSBase. Our results revealed that the original high accuracy reported when the models were trained on CCSBase does not generalize to other chemical spaces. We observed similar results when training on METLIN-CCS and evaluating on CCSBase. Additionally, we demonstrate how an extension of the architecture of GNN (Mol2CCS) including other additional features improves the generalizability of SigmaCCS, particularly for dimers and other uncommon adducts. Lastly, we investigated the application of confidence models and showed how employing them can improve the confidence of the underlying predictions.

Our work highlights that one of the major challenges in the field is lack of data availability. Despite the fact that the release of METLIN-CCS offers several times more data points compared to CCSBase, the lack of generalizability observed is concerning. We believe that our findings impact the usability of any in silico database generated so far, as their predicted CCS values have to be used with caution, especially for uncommon adducts. To mitigate this, we demonstrated how confidence models can be applied to narrow down molecular datasets to high confidence predictions. Another limitation of our evaluation is its restriction in scope, as it mainly focuses on small molecules and specific regions of the chemical space (e.g., METLIN-CCS is based on synthetic structures). Finally the models can only be as good as the underlying experimental data available. We applied filtering strategies similar to those used in previous studies (see Section."Standardization across datasets") to try and mitigate this issue; however, variations in the experimental conditions, such as instrument types, likely introduced some inaccuracies in the CCS values used to train our models.

We foresee several potential avenues for our work. Firstly, the improvements in generalizability shown by our work together with the promising application of a confidence model, can be leveraged to generate in silico datasets with higher quality. Secondly, as new CCS databases are released, or new ML architectures emerge, we expect the scientific community to conduct similar benchmarks in order to verify that an increase in data and an improvement in model architectures indeed improves generalizability. Thirdly, similar to the confidence model application presented in this work, we anticipate that approaches such as generating a distribution of predictions applying Monte Carlo dropout or using ensemble models could be used to assess the confidence of the predictions. Lastly, the confidence model that we trained could be used alongside Monte Carlo dropout to select the prediction with the highest confidence potentially leading to better predictions for more molecules.

### Supplementary Information

Supplementary Material 1.

## Data Availability

Benchmarking scripts and the implemented adaptations for Mol2CCS are released at https://github.com/enveda/ccs-prediction. Here, we include scripts and notebooks to perform grid search, rerun the experiments, process the data, and generate the data splits. All data supporting the conclusions in this article is available at https://zenodo.org/records/11199061.

## References

[1] Baker ES, Hoang C, Uritboonthai W, Heyman HM, Pratt B, MacCoss M et al (2023) METLIN-CCS: an ion mobility spectrometry collision cross section database. Nat Methods 20(12):1836–1837. 10.1038/s41592-023-02078-5 37932399 10.1038/s41592-023-02078-5 PMC10843661

[2] Baker ES, Uritboonthai W, Aisporna A, Hoang C, Heyman HM, Connell L et al (2024) METLIN-CCS lipid database: an authentic standards resource for lipid classification and identification. Nat Metab. 10.1038/s42255-024-01058-z 38802544 10.1038/s42255-024-01058-z PMC11218851

[3] Bemis GW, Murcko MA (1996) The properties of known drugs. 1. molecular frameworks. J Med Chem 39(15):2887–2893 8709122 10.1021/jm9602928

[4] Das S, Tanemura KA, Dinpazhoh L, Keng M, Schumm C, Leahy L et al (2022) *In silico* collision cross section calculations to aid metabolite annotation. J Am Soc Mass Spectrom 33(5):750–759. 10.1021/jasms.1c00315 35378036 10.1021/jasms.1c00315 PMC9277703

[5] Dragos H, Gilles M, Alexandre V (2009) Predicting the predictability: a unified approach to the applicability domain problem of QSAR models. J Chem Inf Model 49(7):1762–1776. 10.1021/ci9000579 19530661 10.1021/ci9000579

[6] Guo R, Zhang Y, Liao Y, Yang Q, Xie T, Fan X et al (2023) Highly accurate and large-scale collision cross sections prediction with graph neural networks. Commun Chem 6(1):139. 10.1038/s42004-023-00939-w 37402835 10.1038/s42004-023-00939-w PMC10319785

[7] Kanu AB, Dwivedi P, Tam M, Matz L, Hill HH Jr (2008) Ion mobility–mass spectrometry. J Mass Spectrom 43(1):1–22. 10.1002/jms.1383 18200615 10.1002/jms.1383

[8] Landrum G. (2016). RDKit: open-source cheminformatics, http://www.rdkit.org/. 10.5281/zenodo.7415128

[9] Li X, Wang H, Jiang M, Ding M, Xu X, Xu B et al (2023) Collision cross section prediction based on machine learning. Molecules 28(10):4050. 10.3390/molecules28104050 37241791 10.3390/molecules28104050 PMC10221386

[10] Luque Ruiz I, Gómez-Nieto MÁ (2018) Study of the applicability domain of the QSAR classification models by means of the rivality and modelability indexes. Molecules 23(11):2756. 10.3390/molecules23112756 30356020 10.3390/molecules23112756 PMC6278359

[11] Ochi S, Miyao T, Funatsu K (2017) Structure modification toward applicability domain of a QSAR/QSPR model considering activity/property. Mol Inf 36(12):1700076. 10.1002/minf.201700076 10.1002/minf.201700076 28815921

[12] Pedregosa F, Varoquaux G, Gramfort A, Michel V, Thirion B, Grisel O, Blondel M et al (2011) Scikit-learn: machine learning in python. J Machine Learn Res 12:2825–2830

[13] Picache JA, Rose BS, Balinski A, Leaptrot KL, Sherrod SD, May JC, McLean JA (2019) Collision cross section compendium to annotate and predict multi-omic compound identities. Chem Sci 10(4):983–993. 10.1039/C8SC04396E 30774892 10.1039/C8SC04396E PMC6349024

[14] Plante PL, Francovic-Fontaine É, May JC, McLean JA, Baker ES, Laviolette F et al (2019) Predicting ion mobility collision cross-sections using a deep neural network: DeepCCS. Anal Chem 91(8):5191–5199. 10.1021/acs.analchem.8b05821 30932474 10.1021/acs.analchem.8b05821 PMC6628689

[15] Preto AJ, Correia PC, Moreira IS (2022) DrugTax: package for drug taxonomy identification and explainable feature extraction. J Cheminform 14(1):73. 10.1186/s13321-022-00649-w 36303244 10.1186/s13321-022-00649-w PMC9609197

[16] Rainey MA, Watson CA, Asef CK, Foster MR, Baker ES, Fernández FM (2022) CCS Predictor 2.0: an open-source jupyter notebook tool for filtering out false positives in metabolomics. Anal Chem 94(50):17456–17466. 10.1021/acs.analchem.2c03491 36473057 10.1021/acs.analchem.2c03491 PMC9772062

[17] Ross DH, Cho JH, Xu L (2020) Breaking down structural diversity for comprehensive prediction of ion-neutral collision cross sections. Anal Chem 92(6):4548–4557. 10.1021/acs.analchem.9b05772 32096630 10.1021/acs.analchem.9b05772

[18] Roy K, Kar S, Ambure P (2015) On a simple approach for determining applicability domain of QSAR models. Chemom Intell Lab Syst 145:22–29. 10.1016/j.chemolab.2015.04.013 10.1016/j.chemolab.2015.04.013

[19] Simonovsky M, Komodakis N. (2017). Dynamic edge-conditioned filters in convolutional neural networks on graphs. Proceedings of the IEEE conference on computer vision and pattern recognition. 3693–3702

[20] Stricker T, Bonner R, Lisacek F, Hopfgartner G (2021) Adduct annotation in liquid chromatography/high-resolution mass spectrometry to enhance compound identification. Anal Bioanal Chem 413:503–517. 10.1007/s00216-020-03019-3 33123762 10.1007/s00216-020-03019-3 PMC7806579

[21] Xie, T., Yang, Q., Sun, J., Zhang, H., Wang, Y., and Lu, H. Large-scale prediction of collision cross-section with graph convolutional network for compound identification.

[22] Xue J, Wang B, Ji H, Li W (2024) RT-transformer: retention time prediction for metabolite annotation to assist in metabolite identification. Bioinformatics. 10.1093/bioinformatics/btae084 38402516 10.1093/bioinformatics/btae084 PMC10914443

[23] Zhang H, Luo M, Wang H, Ren F, Yin Y, Zhu ZJ (2023) AllCCS2: curation of ion mobility collision cross-section atlas for small molecules using comprehensive molecular representations. Anal Chem 95(37):13913–13921. 10.1021/acs.analchem.3c02267 37664900 10.1021/acs.analchem.3c02267

