# Supplementary material for: Evaluating the generalizability of graph neural networks for predicting collision cross section
Source: J Cheminform. 2024 Aug 29;16:105. doi: 10.1186/s13321-024-00899-w (PMC11363525; doi:10.1186/s13321-024-00899-w)
Supplement: Supplementary file 1 — Supplementary Material 1. [file 13321_2024_899_MOESM1_ESM.pdf]

Supplementary File

Supplementary Text 1

Mol2CCS is implemented in Keras and TensorFlow, following the SigmaCCS codebase. Details about the specific version and other libraries can be found in the GitHub repository. GraphCCS is implemented in PyTorch. All three models have been trained on a *c7i.16xlarge* instance deployed in AWS. The instances contain 4th Generation Intel Xeon Scalable processors with 64 CPUs and 128 GBs of RAM memory. In this instance, models require between approximately 20 hours on CCSBase, and 2.5 days on the combined dataset for training 400 epochs.

Supplementary Text 2

The confidence model is implemented as a *RandomForestClassifier*. To identify an optimal set of parameters, we performed grid search using the following parameters: "**n_estimators**": [50, 100, 200, 400], "**max_depth**": [None, 10, 20, 50], "**min_samples_split**": [2, 5, 20]. The input of the model is described in Methods.

When using 5% as a threshold to define the two classes: i) wrong predictions (error larger than 5%), and ii) accurate predictions (error equal or smaller than 5%), we obtain the following number of data points for each class:

- **Predictions on METLIN-CCS when trained on CCSBase:** % of True labels (75.06%), and % of False labels (24.94%)
- **Predictions on CCSBase when trained on METLIN-CCS**: % of True labels (44.64%), and % of False labels (55.36%)

This balanced proportion of labels allows the model to see enough examples of both classes. However, choosing a more/less restrictive threshold influences this proportion and can lead to an unbalanced representation of the two classes, which can be detrimental to the model.

Supplementary Figures

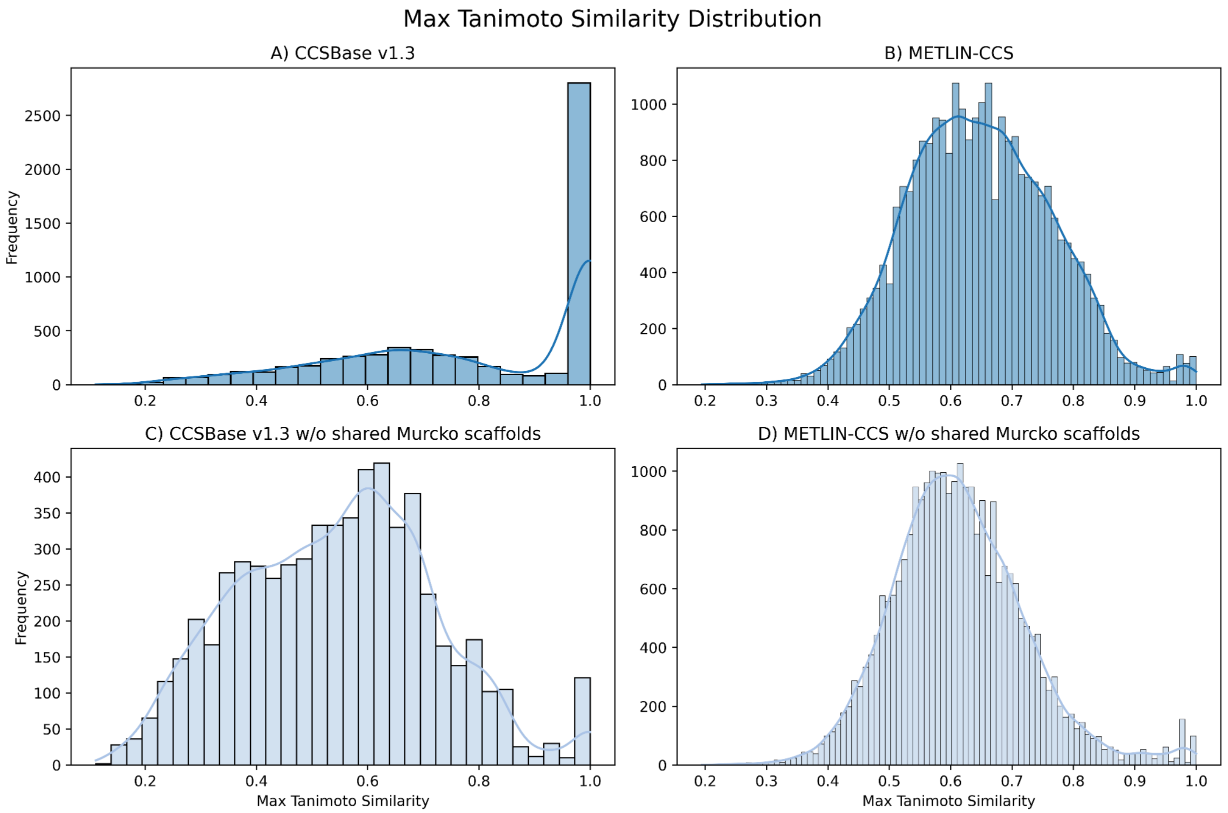

Supplementary Figure 1. Highest Tanimoto similarities for the most similar compound in CCSBase v1.3 (A) and METLIN-CCS (B). (C) and (D) are equivalent distributions but applying a filter for each on molecules that share the same Murcko scaffold (equivalent to applying a Murcko scaffold split). To generate the plot, we calculated the Tanimoto similarity using Morgan fingerprints with radius 2 and the default values in RDKit 2024.03.1 for all pairs (skipping duplicates) in each database independently. The highest Tanimoto coefficient for each molecule is plotted in the distribution above. The distributions without the Murcko scaffold filter show how, for most of the molecules, there is a closely related structure in the dataset in the case of CCSBase with Tanimoto=1, and a fairly similar molecule (0.5-0.75) for METLIN-CCS. Applying the Murcko scaffold filter reduces the number of similar molecules in the case of CCSBase significantly, shifting the distribution to the left.

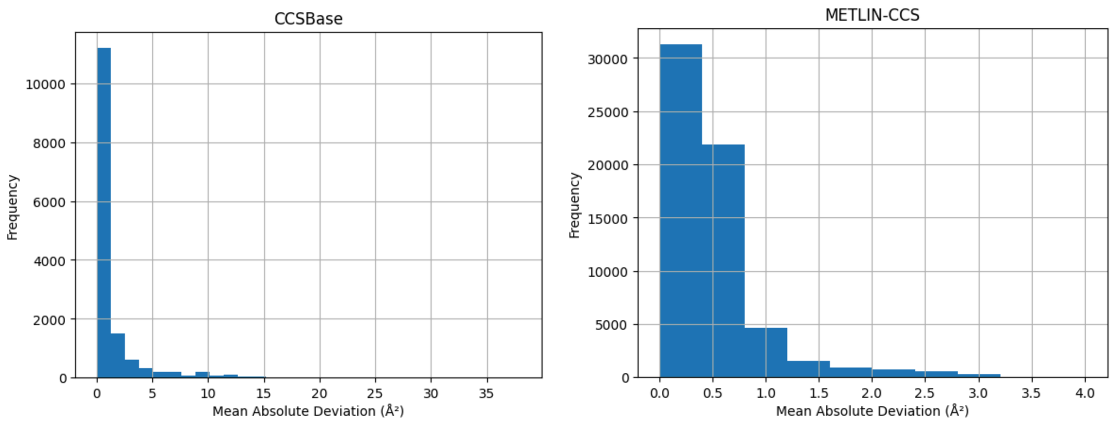

Supplementary Figure 2. Mean absolute deviation for the compounds in the two datasets. Our chosen cutoff (5) is a loose version of the cutoff used in the METLIN-CCS database. They used a cutoff of 2% CV which translates to about 3 mean absolute deviations (right plot). Our chosen cutoff removed the compounds with the most extreme variations while allowing us to keep the majority of the data.

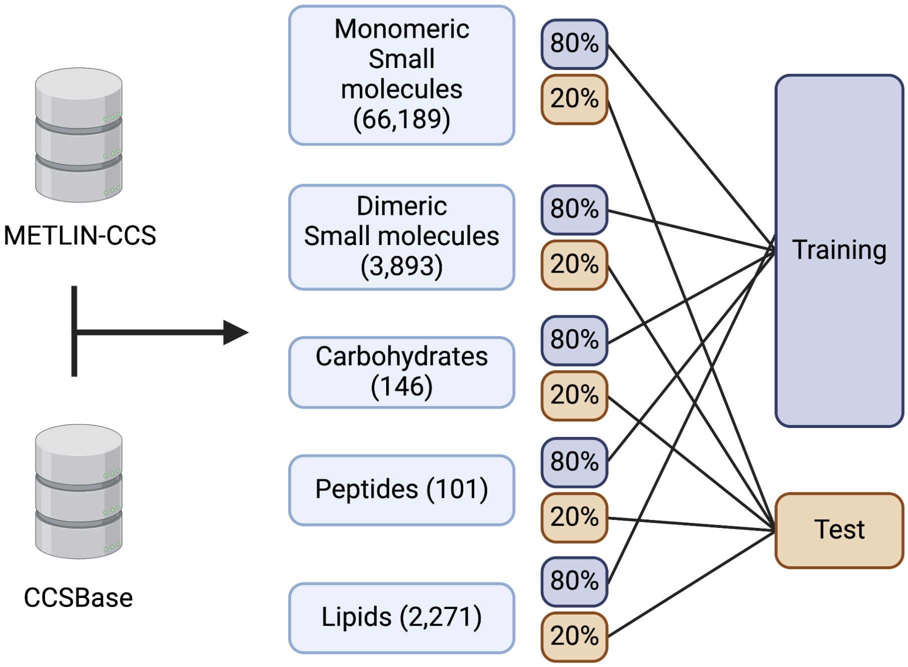

Supplementary Figure 3. Data splitting strategy. Starting with the 10,780 CCS values CCSBase and the 61,862 values for METLIN-CCS, we first grouped them by molecule classes (the numbers in the boxes indicate the number of CCS values per class). Next, we split them based on Murcko scaffolds, generating a training set of approximately 80% of the total data points, and a test set of 20%. Next, we combine these subsets into the final combined train and test set. In the other evaluation setting, in which we train on a single database, we simply

filtered out the CCS data points coming from the other database to similarly obtain a 80/20 split for the specific database of interest (either CCSBase or METLIN-CCS). For instance, when training and evaluating on METLIN-CCS alone, we remove all the carbohydrate, peptide and lipids since none of them are coming from METLIN-CCS. Similarly, after removing the CCSBase subset for small molecules, the remaining METLIN-CCS train and test set remain approximately 80% and 20% of the original database, respectively. That way, the splitting we describe above is maintained and valid for training both databases combined or independently.

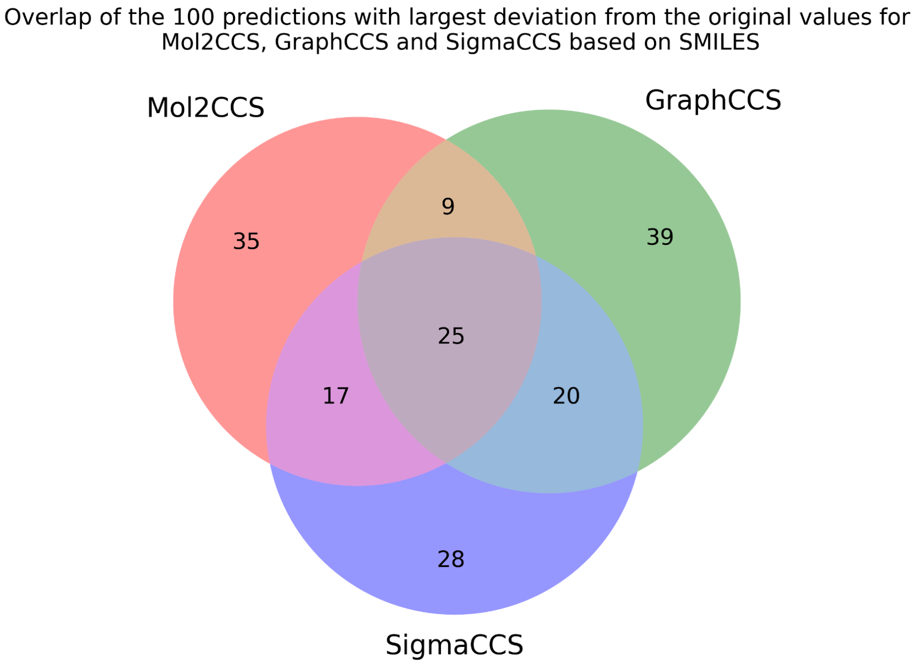

Supplementary Figure 4. Overlap of the 100 predictions with largest deviation from the original values for the three models when training and evaluating on CCSBase. All three models share 25/100 least accurate predictions (outliers). The specific molecules can be explored at the following notebooks from the GitHub repository https://github.com/enveda/ccs-prediction/blob/main/notebooks/exploring_predictions/.

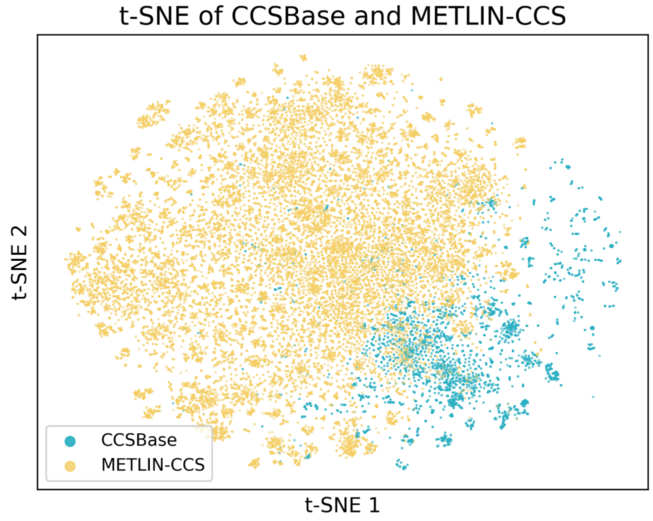

Supplementary Figure 5. Representation of METLIN-CCS (yellow) and CCSBase (blue) by reducing molecular fingerprints into two dimensions using the t-SNE dimensionality reduction method (t-distributed stochastic neighbor embedding). The plot shows how very few data points overlap, meaning that the datasets are structurally very different. We calculated the Morgan fingerprints (256 bits) with radius 2 and the default values in RDKit 2024.03.1.

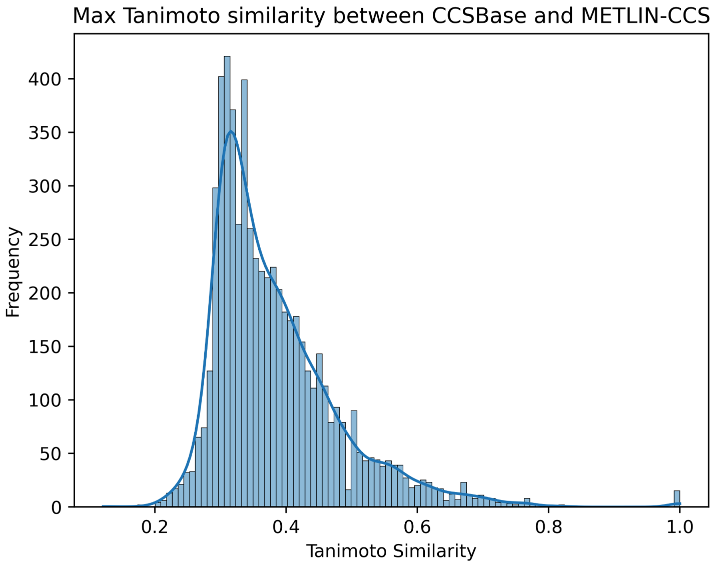

Supplementary Figure 6. Highest Tanimoto similarities for the most similar compound between the two databases (CCSBase and METLIN-CCS). Similar to Supplementary Figure 1, we calculated the Tanimoto similarity using Morgan fingerprints (256 bits) with radius 2 and the default values in RDKit 2024.03.1 for all unique pairs of molecules across both databases. The highest Tanimoto coefficient for each molecule is plotted in the distribution above.

Comparison of Mol2CCS (left) and GraphCCS (middle) predictions, SigmaCCS (right)

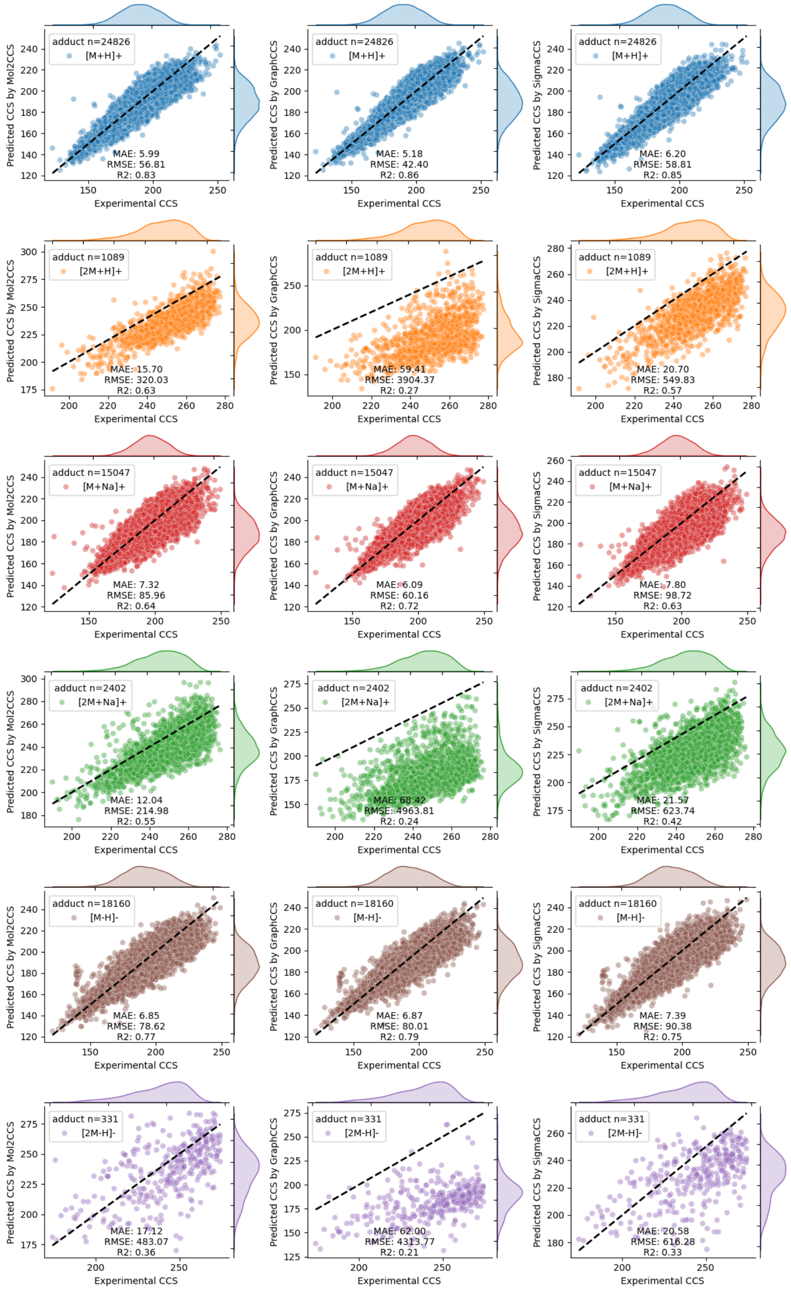

Supplementary Figure 7. Predictions stratified by adduct when training on CCSBase and evaluating on METLIN-CCS across all three models. Each row corresponds to the six adducts present in METLIN-CCS (**Supplementary Table 4).** From these six, the three dimers (violet, green and orange) achieve a poor performance in GraphCCS. The other two models (Mol2CCS and SigmaCCS) show a better performance for these three dimers but they still predict lower values than the experimental ones. This can be attributed to: i) SigmaCCS graph adduct processing which is not explicitly made for dimers, ii) the few examples for dimers

present in the training data (CCSBase). Lastly, for monomers ([M-H]-, [M+H]+, and [M+Na]+), GraphCCS shows a slightly better performance than the other two models.

Comparison of Mol2CCS (left) and GraphCCS (middle) predictions, SigmaCCS (right)

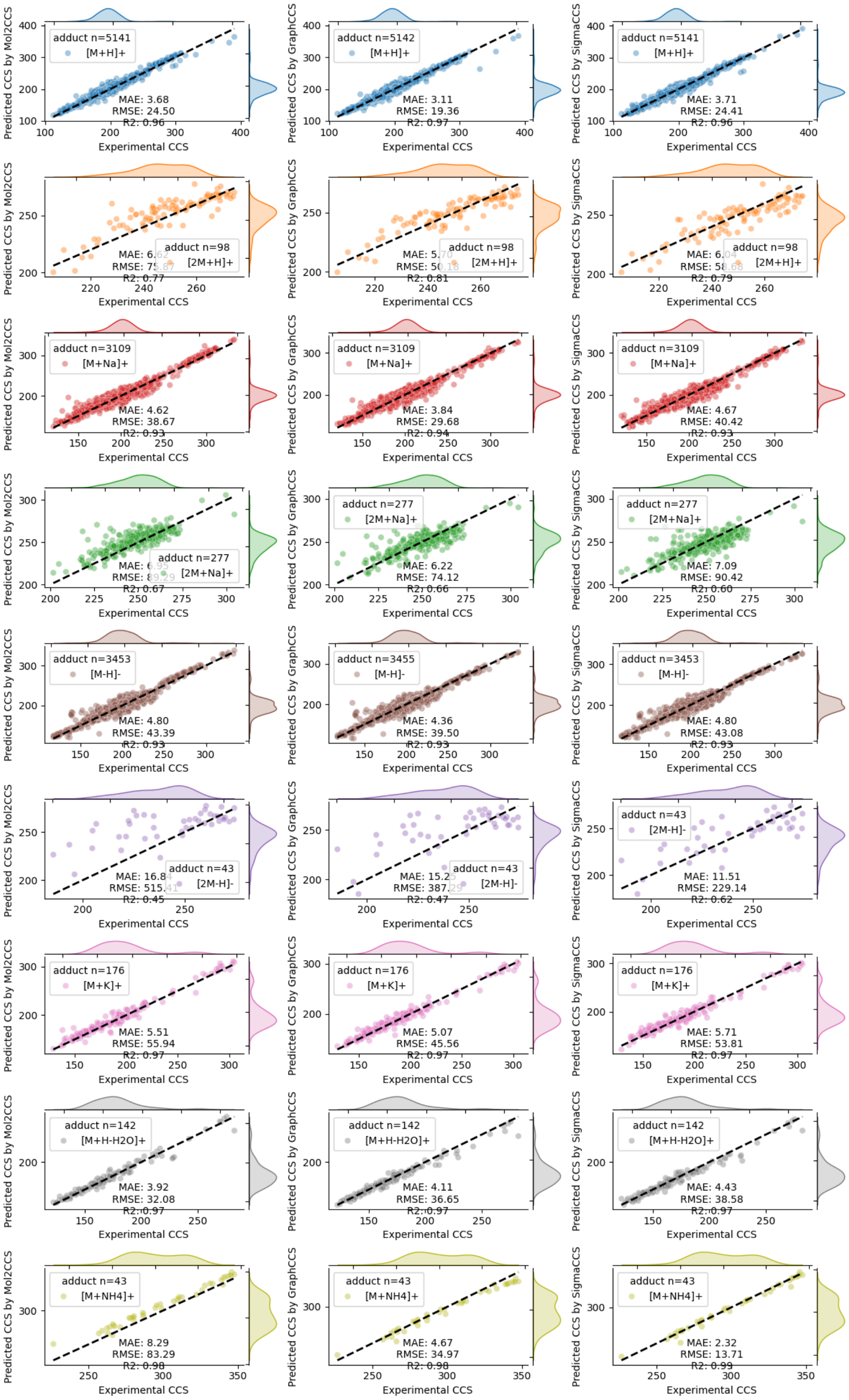

Supplementary Figure 8. Predictions stratified by adduct when training on the combined datasets across all three models. Each row corresponds to the nine adducts present in **Supplementary Table 4.**

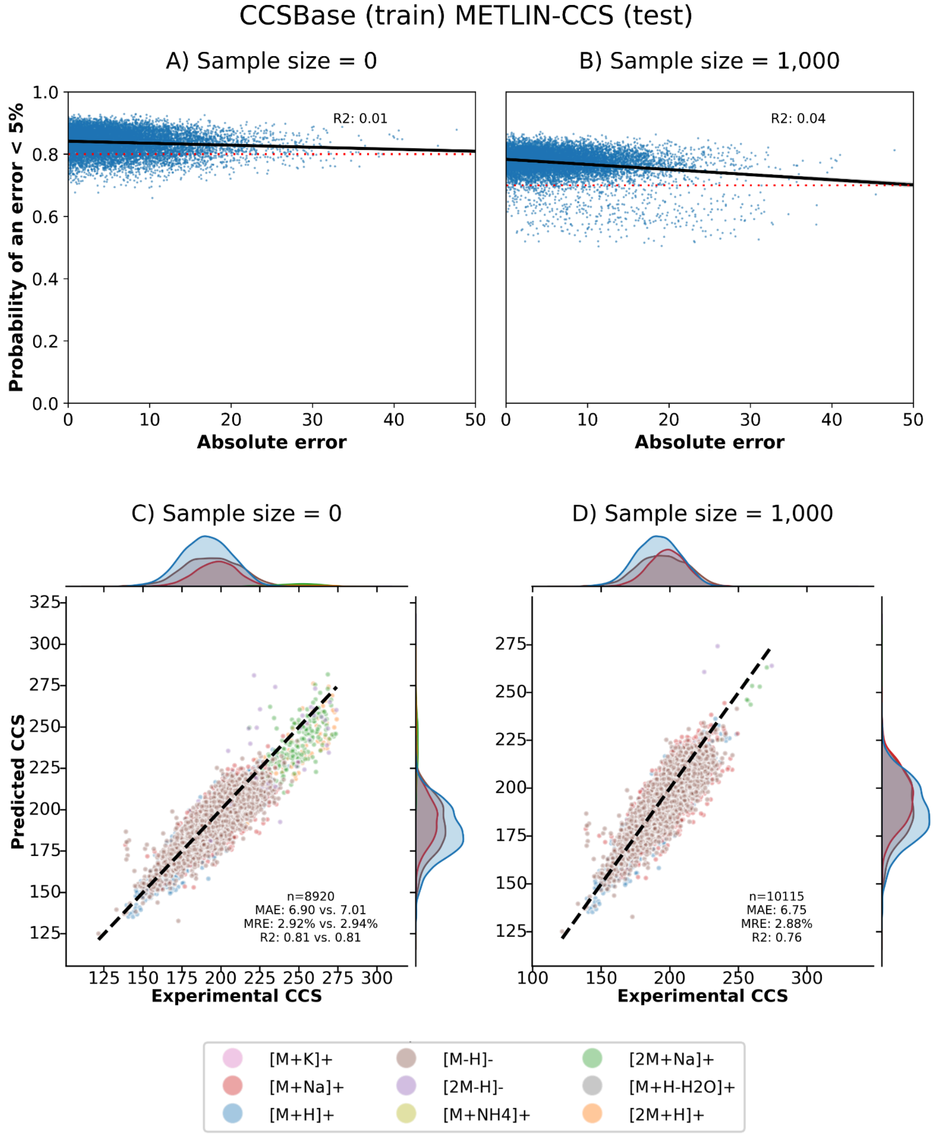

Supplementary Figure 9. Confidence model for CCS prediction model trained on CCSBase and tested on METLIN-CCS (different chemical space setting). A-B) Predicted confidences by the confidence model in the test set vs. absolute error of the Mol2CCS prediction. C-D) Predictions on the high confidence subset generated from the two experiments where the model is trained on one database and evaluated on the other. Graphs A and C are for the confidence model trained only on data from CCSBase. Graphs B and D show results for the confidence model trained on CCSBase data with an additional 1,000 data points from METLIN-CCS (that are structure disjoint from the METLIN-CCS test dataset).

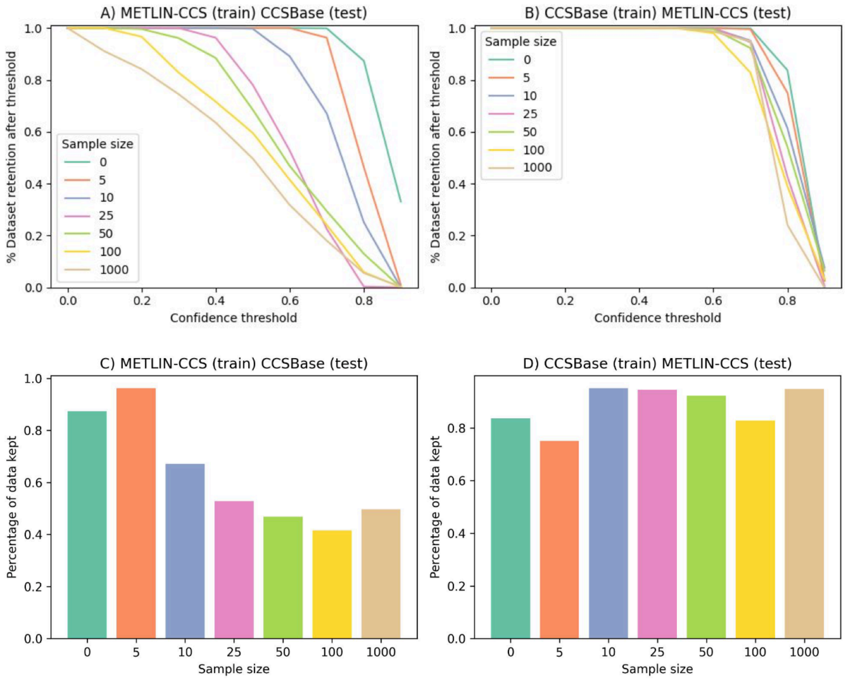

Supplementary Figure 10. A-B) Percentage of dataset remaining after confidence thresholding for various confidence thresholds. C-D) Percentage of dataset remaining for thresholds used in Section 3.4 Each confidence model is trained with the test set of the database used to train the CCS prediction model (METLIN-CCS for the left graph). In addition to this data, a small scaffold-disjoint sample of data from the domain of the confidence model test set (CCSBase for the left graph) is included in the training dataset for the confidence model. As more data from this domain is included in the confidence model training set, the confidence model generally gets less confident meaning that more data is filtered out for higher thresholds. For graph C, the thresholds used were 0.8, 0.7, 0.7, 0.6, 0.6, 0.6, and 0.5, respectively (starting with a sample size of 0). For graph D, the thresholds were 0.8 for sample sizes 0 and 5 and 0.7 for the rest. All of the thresholds were determined using the method described in **Section 2.7**.

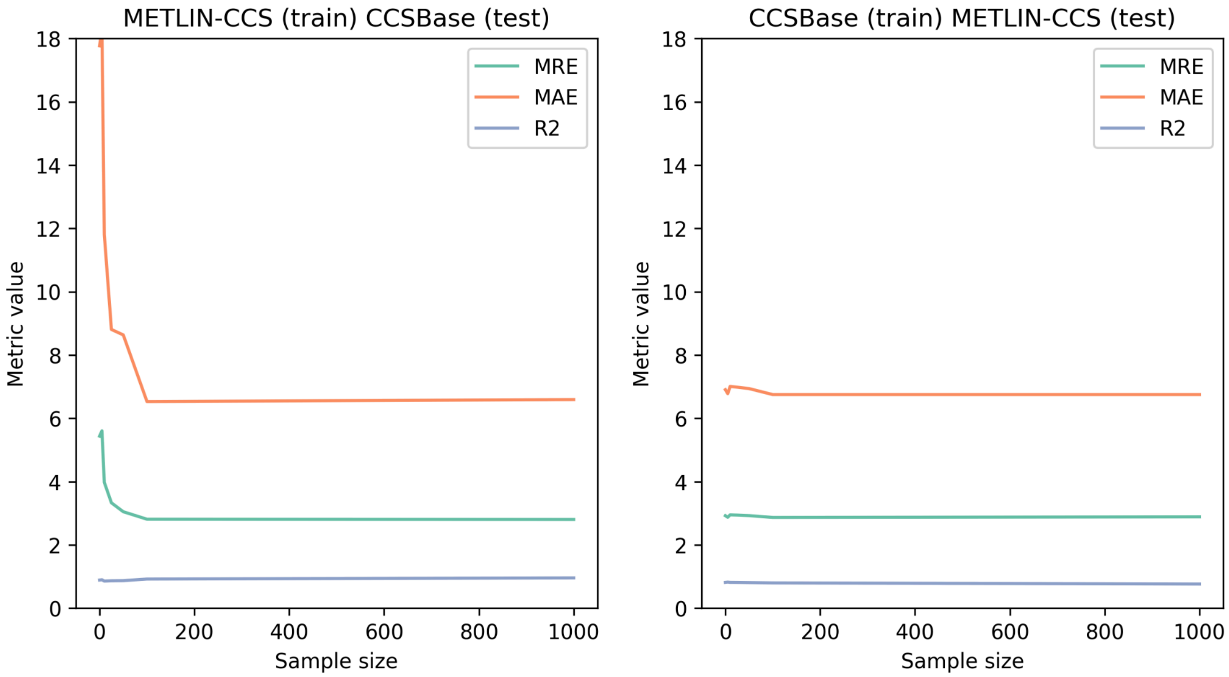

Supplementary Figure 11. Metrics for confidence model as more data is added to the confidence model training set from the same domain as the test set (still scaffold disjoint from the test set). As more in-domain data is added, the metrics improved for both models, though more significantly for the model trained on METLIN-CCS.

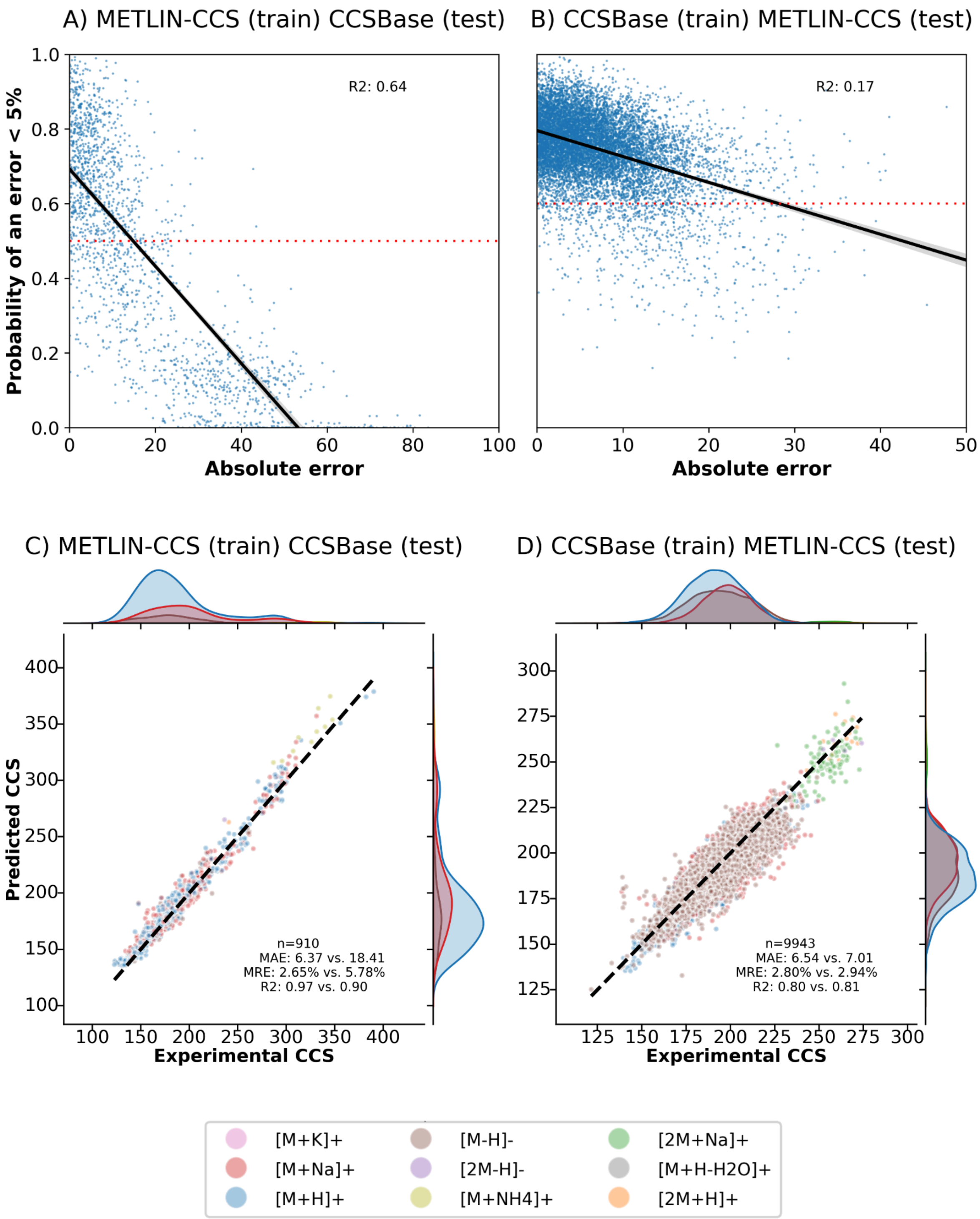

Supplementary Figure 12. A-B) Predicted confidences by the confidence model in the test set vs. absolute error of the Mol2CCS prediction (similar chemical space setting). C-D) Predictions on the high confidence subset generated from the two experiments where

the model is trained on one database and evaluated on the other. The metrics for this subset of high confidence predictions are compared against the full test set.

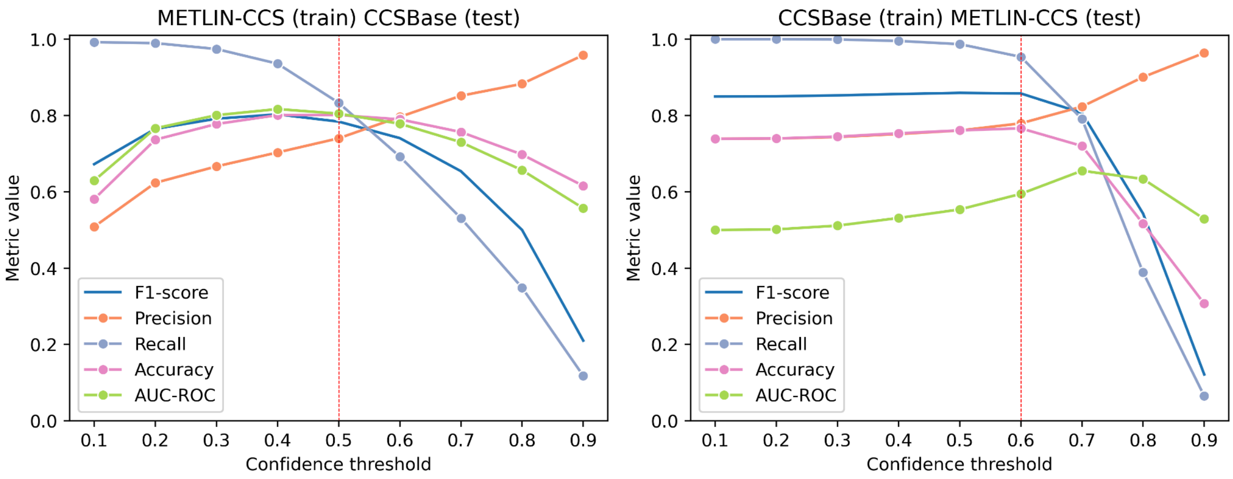

Supplementary Figure 13. Evaluation of the confidence model using different thresholds on the validation set (X-axis). The evaluation measures the F1-score, precision, recall, accuracy and AUC-ROC based on the subset of the dataset after applying the threshold. In other words, for each of the 10 thresholds plotted (e.g., 0.1, 0.2, 0.3, etc), we remove the predictions with confidence lower than the given threshold and subsequently evaluate the reported metrics. For this evaluation, probabilities below 0.5 are classified as “non accurate” predictions (i.e., the predicted CCS values are deviated from the original CCS value by 5% or more) and probabilities above 0.5 are considered as “accurate” predictions (i.e., the predicted CCS values are deviated from the original CCS value by less than 5%) (**Supplementary Figure 11A-B**). Each of the reported metric evaluates the agreement between these assigned/predicted labels by the confidence model and the actual labels based on the observed difference between the predicted and the experimental CCS value. For example, suppose that a model (e.g., Mol2CCS) predicts a CCS value of 106, and the experimental value is 100, and the confidence model gives that prediction a probability of 0.4 (“non accurate” prediction label). This prediction by the confidence model would be considered accurate given the 5% threshold used since the difference between the predicted CCS value and the experimental one is larger than 5%, and the probability is lower than 0.5. By comparing the predictions and the actual labels, we can derive the metrics reported in the figure.

Supplementary Tables

Type	Attribute
Atom	One-hot encoding of the atom element

Atom	One-hot encoding of atom's degree
Atom	Atom radius
Atom	One-hot encoding indicating whether the node is present on a ring or not
Atom	Atom mass
Atom	3D coordinates
Atom	Atom charge
Atom	One-hot encoding indicating whether the atom is chiral or not
Atom	TPSA (surface area)
Atom	ASA (surface area)
Atom	Crippen contribution
Edge	One-hot encoding of the bond type
Edge	One-hot encoding indicating if the bond in conjugated or not
Edge	One-hot encoding indicating if the bond is present in a ring or not
Edge	One-hot encoding indicating the type of stereo information of the bond (*GetStereo*)
Edge	One-hot encoding with bond direction (*getBondDir*)

Supplementary Table 1. Node and edge attributes. These node and edge attributes are used in their respective matrices to train the GNN module in Mol2CCS **(see Figure 1A)**.

**Parameter**	**Range evaluated**
Batch size	[16, **32**, 64]
Dropout rate	[0.0, **0.1,** 0.2, 0.5]
Learning rate	0.0001

Supplementary Table 2. Hyperparameter grid search used to find the optimal parameters to train the model. We highlight in bold the best parameters found.

**Molecule type**	**CCSBase**	**METLIN-CCS**
Small molecules	4,376	27,629
Lipids	1,535	0
Peptides	97	0

Carbohydrates	67	0

Supplementary Table 3. Types of molecules in each database. While METLIN-CCS exclusively consists of small molecules, CCSBase contains other molecule classes such as lipids, carbohydrates and peptides. However, while the chemical similarity between the molecules in CCSBase is extremely high, METLIN-CCS contains a broader and more diverse set of small molecules **(Supplementary Figure 1)**.

Adduct	CCSBase	METLIN-CCS
[M+H]+	3,998	24,826
[M-H]-	1,751	18,160
[M+Na]+	2,591	15,047
[M+K]+	1,111	0
[M+NH4]+	320	0
[M+H-H2O]+	938	0
[2M-H]-	32	331
[2M+Na]+	29	2,402
[2M+H]+	10	1,089

Supplementary Table 4. Adduct types in each database. CCSBase contains examples of all nine adducts evaluated in this work. However, the three dimers are poorly represented in the database. In the case of METLIN-CCS, there is no data for 3 of the 9 adducts ([M+Na]+, [M+K]+, and [M+NH4]+).
